# Initial Psychometric Evidence of Physical Inactivity Perceived Experience Scale (Pipes): COVID-19 Pandemic as a Pilot Study

**DOI:** 10.3389/fpubh.2022.819052

**Published:** 2022-03-22

**Authors:** Noomen Guelmami, Nasr Chalghaf, Amayra Tannoubi, Luca Puce, Fairouz Azaiez, Nicola Luigi Bragazzi

**Affiliations:** ^1^Postgraduate School of Public Health, Department of Health Sciences (DISSAL), University of Genoa, Genoa, Italy; ^2^Group for the Study of Development and Social Environment (GEDES), Faculty of Human and Social Science of Tunis, Tunis, Tunisia; ^3^Department of Human and Social Sciences, Higher Institute of Sport and Physical Education of Kef, University of Jendouba, Jendouba, Tunisia; ^4^Department of Human Sciences, Higher Institute of Sport and Physical Education of Sfax, University of Sfax, Sfax, Tunisia; ^5^Department of Neuroscience, University of Genoa, Genoa, Italy; ^6^Laboratory for Industrial and Applied Mathematics (LIAM), York University, Toronto, Toronto, ON, Canada; ^7^Department of Mathematics and Statistics, York University, Toronto, ON, Canada

**Keywords:** COVID-19, physical inactivity, fear, perceived stress, factorial invariance, scale validation

## Abstract

**Aims:**

Our study aimed to develop a two-factor self-administered orthogonal questionnaire to assess the experience of perceived physical inactivity, to test its psychometric properties, to confirm its relationships with fear of COVID-19, and finally, with perceived stress during the pandemic.

**Methods:**

A total of 481 Tunisian subjects collected in several cities, aged from 16 to 67 years with a mean age = 32.48 ± 9.46, and of both sexes participate in our study with (male: 51.8%) and (female: 48.2%), divided according to the level of study into three categories. All subjects voluntarily answered the PIPES questionnaire, the IPAQ scale, the COVID-19 fear scale and the PSS-10 test.

**Results:**

The results of the exploratory and confirmatory factor analysis supported the robustness of the tool measure. In addition, examination of configurational, metric, scalar, and strict invariance supported the equivalence of the structure by gender and educational level. Concurrent validity was established by the positive association of a negative perception of physical inactivity with scores measured by the IPAQ scale and a negative association with scores of COVID-19 fear and perceived stress. Whereas, a positive perception of physical inactivity from the COVID-19 scale was negatively associated with the IPAQ and positively associated with fear of COVID-19 and perceived stress.

**Conclusion:**

The PIPES-10 scale can be used to measure the perception of physical inactivity in different situations.

## Introduction

The benefits of physical activity and exercise on physical and mental health, as well as the negative impacts of physical inactivity, have been well documented in the scientific literature for both adults and children (1–4). For physical health, many researchers have highlighted the role of physical inactivity in the prevalence of various pathologies. Several longitudinal and cross-sectional studies for different age groups and in both sexes report evidence of the benefits of exercise on the prevention and treatment of several diseases related to the cardiovascular systems (5–9), respiratory (10, 11), immune (12), diabetes (13, 14), neurogenic diseases (15, 16), cancer (17), obesity (18), and many other diseases.

Similarly, in human psychology, numerous studies have confirmed strong associations between physical inactivity and various negative behaviors and psychological parameters such as stress, depression and anxiety (19–22). Moreover, in contemporary sociology, a plethora of work has established links and explanatory models for the benefits of physical activity with several social factors (23–25).

As a result, findings have been reported by physicians, biologists, psychologists, and sociologists on the need to promote exercise and regular physical activity. Many researchers cite sedentary behavior and physical inactivity as a major risk factor that increases lethality rates in contemporary societies.

Despite all of these substantial changes, lifestyles across countries vary and physical inactivity in many countries is likely to persist to become an international pandemic in 2012. Globally, physical inactivity is presented as the greatest public health problem of the twentyfirst century (26) and the fourth leading cause of death (27), its economic consequences are also severe (27). Physical inactivity is currently considered a pandemic that has become a major concern for several international organizations, such as the World Health Organization (WHO) and the Centers for Disease Control and Prevention (CDC). Sustained physical inactivity and sedentary behavior are generally associated with poor physical and mental health and increased risk of mortality (6, 7, 28–30).

Physical inactivity across multiple populations and countries is increasingly worsening, particularly during the COVID-19 pandemic, which was a particular global experience characterized by specific measures that imposed containment, restrictions on travel between countries, and even habitual travel within cities of the same country (31, 32). Indeed, during the COVID-19 pandemic, several studies have reported a significant increase in physical inactivity would be evident due to the requirements of self-isolation and quarantine in addition to the curfews. In other words, in several countries, the rapid growth of the COVID-19 pandemic has forced governments to put in place a curfew (33), stoppages, or restrictions on movement.

These government decisions were accompanied by a decrease in physical loads at work, the cessation of schooling, the suspension of all sports activities and competitions, in addition to the closure of several places of physical activities such as sports centers, sports halls, amusement parks, municipal stadiums and private fields (31).

Under these specific conditions often accompanied by fear of COVID-19, stress, anxiety, and depression (34–37), physical inactivity could significantly increase mortality rates in several populations (38–40) and particularly in patients and vulnerable groups such as obese, diabetic, hypertensive, and cancer patients. In this regard, Stanton et al. (41) reported during the pandemic an increase in physical inactivity associated with increased depression, anxiety, and stress.

This increase in inactivity can be dramatic in many populations and deserves a measurement tool specific to this environment. Indeed, several physical activity measurement tools have been developed over time to target the perception of physical activity (42). As an example, Fox and Corbin (43) developed the physical self-perception profile based on self-esteem theories. In another work, Kerner and Kalinski (44) developed a measure for young people through attitudes, beliefs, perception of control, and intention to engage in leisure-time physical activity. And Salvador et al. (45) study who develops the “Perception of the environment and leisure-time physical activity in the elderly”. However, these scales were not general (for example, focused on specific physical activities such as leisure activities), never considered physical inactivity, and were mostly developed for specific populations. To the best of our knowledge, there is no measurement scale which attempted to measure the perception of the experience of physical inactivity in relation to a pandemic environment and adjacent specific measures. It is therefore very important to construct a standard tool that can assess this, especially in a phase of awareness of the importance of having a healthy body with strict measures such as movement restriction and containment. The objective of this paper is to develop a self-administered questionnaire that measures the perception of the experience of physical inactivity, to test its psychometric properties and to confirm the relationships between the perception of the experience of physical inactivity with the fear of COVID-19, and the perceived stress during the pandemic.

## Materials and Methods

### Instruments

The questionnaire items were developed based on an in-depth analysis of specific literature and expert feedback. Before making decisions about scale development, we considered several models and theories in the context of physical exercise, such as the behavioral epidemiological framework advocated, which looks at the link between behaviors and health and disease (46), the theory of planned behavior which has been dominant for years (47) and the Health Belief Model (48). After examining and criticizing the first two theories [e.g., (49)], we are committed to exploiting the latter model for those overarching considerations that could classify attitudes toward activity and physical inactivity at my time.

Indeed, current research based on the Health Belief Model considers that verbal responses regarding attitudes toward physical activity must include expressions about the intention to be physically active or not (50).

As a result, a self-reported measure of attitudes applied to physical activity must include two constructs: one construct centered on expressions that promote physical activity, while the second construct is interested in evaluating a positive attitude with regard to physical inactivity.

In the process, the theoretical design of the first concept of perceived experience of physical inactivity (PIPES1) avoided conceptualizing the construct from a perspective that considers the specific effects of physical activity on physical health factors, mental health or social interactions. This allows for a general conceptualization that can encompass all the factors mentioned without detailing the perceived benefits that are detailed in the non-verbal model of attitudes.

For the second concept, which is also the negative perception of physical inactivity, a general construct was established. This construct was generally related to barriers to practice without detailing the reasons for physical activity inability, such as time required, lack of adequate infrastructure, lack of safety, physical disability. This choice made it possible to measure the concept in a global way. As a result, the cognitive and affective response categories of the Health Belief Model (50, 51). Non-response to long-form questionnaires in the health context [see: (52–54)], the cost and time of administration [for example, (55)], led us to limit ourselves to a reduced number of items.An initial 12-item instrument was generated to measure the two constructs with 6 items for each. Next, the tool was subjected to a review by two experts in physical activity behavior and two university professors specializing in Arabic and English. The thorough review by the panel of experts recommended the elimination of two items that could present ambiguities in the responses (their link with the time factor). The two items “I consider that doing physical activity is a waste of time” and “I consider that the moment of doing physical activity is essential” were eliminated.

The final version led to the generation of 10 items that were retained to measure two orthogonal constructs.

The two factors Positive and Negative perceptions of physical inactivity were then measured with five-items for each of them. A five-point Lickert scale was favored for collecting responses as follows: strongly disagree (1 point), disagree (2 points), neutral (3 points) agree (4 points), totally agree (5 points).

### Physical Activity Level

The level of physical activity was assessed by the official Arabic abbreviated version of the IPAQ (56).

This measure of physical activity has established good psychometric properties in several populations (57–59).

The seven-item IPAQ-C records self-reported physical activity over the past seven days. Responses were converted into minutes of metabolic equivalent tasks per week (MET-min/week) according to the IPAQ scoring protocol: the total number of minutes in the last seven days spent in vigorous activity, moderate-intensity activity, and walking was multiplied by 8.0, 4.0, and 3.3, respectively, to create MET scores for each activity level. MET scores in the three sub-components were added to indicate overall physical activity. Levels of physical activity were also categorized into three categories: small, moderate, and high, according to the scoring system provided by the IPAQ. In this research, we consider this classification of three categories to make a judgment on practicing physical activity.

### COVID-19 Fear Scale

An adapted Arabic version of the COVID-19 scale was applied to illustrate the fear of COVID-19 (60). Reliability and validity were inspected through 693 Saudi participants and confirmed the unique construct of the tool. The internal Arabic consistency was satisfactory (α = 0.88), with a healthy concomitant validity indicated by significant and positive correlations with the HADS anxiety scale (*r* = 0.66).

The initial scale was examined with 717 Iranian participants. After evaluation, using both the classic test theory and the Rasch model, the properties of the scale were satisfactory: internal consistency (α = 0.82) and test-retest reliability (ICC = 0.72) were acceptable.

Good psychometric properties similar to the original instrument have been proven in a Turkish version, an Italian adaptation, and a model built in Bangladesh.

The Turkish version reveals its robustness of measurement and the one-dimensional nature of the tool in 1,304 participants, aged 18 to 64, in 75 cities across confirmatory factor analysis, Item Response Theory, convergent validity, and internal consistency (Cronbach's α, McDonald's ω, Guttmann's λ6, and composite reliability). Likewise, Cronbach's alpha of the Italian version was 0.871 and displayed high-quality reliability. The results of the confirmatory factor analysis of the Bangladeshi version confirmed the unidimensional factor structure of the scale and very good internal reliability.

### Perceived Stress Scale

To assess perceived stress, the version of 10 items in Arabic validated by Almadi et al. (61) was used. The instrument is adapted from the initial scale of Cohen et al. (62), which is the most widely used scale in the world to assess perceived stress as two first-order components, assessed on a Lickert scale of 5 points.

The psychometric properties of the initial scale and the different adaptations have confirmed their measurement robustness in several studies for different populations (63–65).

### Data Collection

Data were collected with a total of 481 subjects aged between 16 and 67 years old with a mean age (M = 32.48, SD = 9.46), over a three month period (March, April, May 2020) in two ways: (1) on work sites, shops, and administrations in several Tunisian cities (*n* = 257, 53.4%) and (2) by a questionnaire sent by email to several contacts (*n* = 224, 46.6%).

Study participants consist of males (*n* = 249, 51.8%) and females (*n* = 232, 48.2%). The distribution of the study level was (34.7%) subjects who had a basic study level (<10 years; *n* = 167), 34.5% who had completed their secondary school studies (*n* = 166), and 30.8% who had a higher level (*n* = 148). No significant difference in the χ^2^ test was demonstrated according to the three variables: age (*p* = 0.44), method of administration (*p* = 0.13) and level of study (*p* = 0.49).

### Statistical Analysis

Preliminary data analysis was performed to examine the quality of the data collected and to inspect if there are any anomalies or missing boxes. Missing data were excluded from the analysis. Subsequently, tests for univariate (Skewness and Kurtosis) and multivariate normality by the Mardia coefficient, were performed. Also, descriptive statistics for each variable were done.

Exploratory factor analysis was performed by the Unweighted Least Squares method with Direct-Oblimin rotation and Kaiser Normalization.

The reliability of the instrument was examined simultaneously by Cronbach's α coefficient, McDonald's ω coefficient, and the composite reliability coefficient CR calculated from the Factor Loading set and the error variances.

The questionnaire structure of the entire population was carried out by confirmatory factor analysis (CFA). Several indices of the CFA were retained to examine the model: (1) the χ2; (2) χ^2^/DF, (3) the comparative fit index (CFI); (4) Tucker-Lewis index (TLI); and (5) the Root Mean Square Error of Approximation (RMSEA).

The recommendations of Hu and Bentler (66) suggested values >0.95 for CFI and TLI and RMSEA values of <0.08 for reasonable fits. The equivalence of the two-factor and 10-item model across the three variables gender, study level, and the method of administration was achieved through confirmatory multi-group factor analysis for four models of invariance tested successively.

The first invariance tested is the Configural Invariance. This step is designed to test whether the indicators have the same free and fixed load pattern across groups.

Once the Configural invariance is confirmed, the increasing comparisons from one model to the next, by imposing a more restrictive level of invariance between the samples of nested model configuration, are tested according to a complexity hierarchy with constraints.

The second step, called the metric invariance test, is to ensure that the different groups answer the questions similarly or equivalently. The technical examination of metric invariance consists of showing that factor loadings are similar to the factors of the measurement scale in the groups.If the metric invariance is assured, the next step is to evaluate the scale invariance. Scalar invariance means that the item interceptions are equivalent between the groups, which means that the group differences in the item mean should give differences in the means of the factors constructed by these indicators. In other words, this implies that subjects with the same value in a factor should have equal values of the indicators.

The last step is to test the residual invariance or the similarity of errors across groups. Residual invariance means that the sum of the specific variance (variance of the item that is not shared with the factor) and the measurement error variance is similar for the different groups.

The Chi-square difference between models was performed to test for invariance in structural equation models. Also, the difference in CFI which must be <0.01 was retained as a criterion to establish the factorial invariance.

Concurrent validity was tested by examining the association between the two instrument factors and the three scales: the IPAQ scale, the COVID-19 Fear scale, and the Perceived Stress Scale *via* a Pearson correlation.

Statistical analyzes were performed using IBM SPSS Software version 26.0 for Windows. While the examination of the different factor structures was carried out by IBM SPSS Amos Software for Windows version 23 (See Table 1).

**Table 1:** Mean (M), SD, confidence interval 95%, skewness (S), kurtosis (K), and factor loadings (λ) by item.

**Items**	**Mean**	**SD**	**Skewness**	**Kurtosis**	**Lamda**
Item1	2.88	1.36	0.09	−1.14	0.903
Item3	2.98	1.39	0.01	−1.26	0.829
Item5	3.02	1.39	−0.02	−1.24	0.797
Item7	2.99	1.41	0.03	−1.28	0.853
Item9	2.99	1.36	−0.02	−1.21	0.896
Item2	2.65	1.24	0.24	−0.97	0.831
Item4	2.63	1.25	0.28	−0.98	0.845
Item6	2.63	1.24	0.28	−0.89	0.847
Item8	2.60	1.28	0.37	−0.92	0.831
Item10	2.62	1.28	0.24	−1.01	0.842

We retained the significance levels for a value of *p* < 0.05 for all statistical analysis.

### Ethics Statement

This work has received approval from the ethics committee of the “Research Unit, Sportive Performance, and Physical Rehabilitation, High Institute of Sports and Physical Education, Kef, University of Jendouba, Jendouba, Tunisia” and received ethical clearance from the UNESCO Chair “Health Anthropology Biosphere and Healing Systems,” “University of Genoa, Genoa (Italy),” the “Higher Institute of Sport and Physical Education of Kef, Kef (Tunisia),” and the “Higher Institute of Sport and Physical Education of Sfax, Sfax (Tunisia).” The proposal has been also approved by the “Jendouba University” Ethics Committee and was undertaken following the legal standards of the Helsinki declaration in 1964 and its corresponding amendments.

## Results

The statistical analysis began by calculating descriptive statistics (means and standard deviations) and inspecting the distributions of the 10 items of the questionnaire. The normality of each item was considered through the examination of Kurtosis and Skewness.

The results of the exploratory factor analysis by the Unweighted Least Squares method using a Direct-Oblimin rotation with Kaiser Normalization resulted in the extraction of two factors that explain 72.17% of the total variance.

The 10 items were subjected to exploratory factor analysis using the Unweighted Least Squares method. The adequacy of the sampling is supported by the index KMO = 0.92 (Kaiser-Meyer-Olkin which measures the quality of the sampling and the quality of the correlation matrices by the significant Bartlett test (x^2^ = 607,132, *p* < 0.001).

### Internal Consistency

Instrument reliability was examined by both Cronbach's α coefficient, McDonald's ω coefficient, and the composite reliability coefficient CR calculated from a Factor Loading set and the error variable (derived from the initial model output of AMOS Software for the whole population).

Table 2 denotes the reliability coefficients for the two instrument factors.

**Table 2:** Reliabilities of the PIPES-10.

**English items**	**Factors**	**McDonald's** **ω**	**Cronbach's** **α**	**Composite reliability**
1. The lack of physical and sports activities is understandable to me.	PIPES1	0.933	0.933	0.887
2. Reducing or discontinuing my physical and athletic activity is worrying to me.				
3. Not being physically active or exercising is something I do not easily accept.				
4. The lack of physical and sports activities has several negative repercussions.				
5. I consider the decision not to engage in physical and sports activities to be completely unsatisfactory.				
6. I canceled many of my physical moves and activities with complete conviction		0.906	0.905	0.881
7. Physical and sporting activities should be discontinued.				
8. I find that reducing physical and athletic activity is necessary.				
9. I am fully convinced that I should not be physically or physically active.				
10. Not doing sports and physical activities has a negative repercussion.				

To test the factorial invariance of the designed tool, several successive models were tested. The specification of the links, variances, and covariances of these models gradually becomes more severe until the complete invariance of the model is demonstrated (67, 68).

The results of the configuration invariance by gender indicated that the model fit was adequate, χ^2^ (66) = 137.28; *p* < 0.001; CFI = 0.9842; TLI = 0.975; and RMSEA = 0.047. These values demonstrate that women and men conceptualize the two perception constructs of physical activity similarly (See Table 3).

**Table 3:** Factorial invariance comparison.

**Invariance**	**X^2^(df)**	**df**	**CFI**	**TLI**	**RMSEA**	**Δ**	**ΔX^2^**	**Δdf**	**p**	**ΔCFI**
M.0	91.3	33	0.985	0.980	0.061					
**Gender**										
Configural (M1)	137.28	66	0.982	0.975	0.047					
Metric (M2)	146.38	74	0.983	0.979	0.043	M2-M1	9.10	8	0.334	0.001
Scalar (M3)	155.75	86	0.982	0.981	0.045	M3-M2	9.37	12	0.670	−0.001
Strict (M4)	175.97	98	0.980	0.982	0.041	M4-M3	20.22	12	0.063	−0.002
**Study Level**										
Configural (M5)	174.89	99	0.981	0.973	0.040					
Metric (M6)	192.94	119	0.981	0.978	0.036	M6-M5	18.05	20	0.584	0.000
Scalar (M7)	220.52	139	0.979	0.980	0.035	M7-M6	27.58	20	0.120	−0.002
Strict (M8)	234.71	163	0.982	0.985	0.031	M8-M7	14.19	24	0.942	0.003
**Administration** **of the Questionnaire**										
Configural (M9)	131.47	66	0.983	0.977	0.046					
Metric (M10)	133.85	74	0.985	0.981	0.041	M10-M9	2.38	8	0.967	0.002
Scalar (M11)	140.23	86	0.986	0.985	0.036	M11-M10	6.38	12	0.90	0.001
Strict (M12)	154.54	98	0.985	0.987	0.035	M12-M11	14.31	12	0.281	−0.001

*All values of X^2^ were significant at p < 0.001*.

For the metric invariance tests, a non-significant statistical difference χ^2^ was demonstrated [Δχ^2^ (8) = 9.10; *p* = 0.334]. As a result, participants from different groups respond to items in the same way, that is, the strengths of the relationships between specific scale items and their constructed factors are the same from group to group.

The scalar invariance provided a non-significant statistical difference χ^2^ [Δχ^2^ (12) = 9.37; *p* = 0.670]. As such, the results indicated that the equal interception constraints kept the solution fit. Assuming the equivalence of the item intersections, we were able to compare the latent means. This implies that the factor loads and their means are equivalent to women and men (See Table 3).

To test for strict factor invariance, equal constraints were imposed on the factor loads, the intersections, residuals, variances, and covariances. The results for Strict invariance across the three variables, the gender, the level of study, and the methods of administration showed non-significant Δdf with ΔCFI that are <0.01. This demonstrates the strict invariance of the tool for the different groups.

For the strict factorial invariance, a statistical difference χ^2^ [Δχ^2^ (12) = 20.22; *p* = 0.063] and a ΔCFI = −0.002 were highlighted. This result indicates that our model is gender invariant (See Table 3).

The tests of configural invariance according to the study level and the method of administration of the questionnaire proved the robustness of the factorial structure through the two models M5 and M9 respectively. Indeed, the results of the configural invariance for the M5 model presented a value of X^2^ (99) = 174.89, CFI = 0.981, TLI = 0.973 and RMSEA = 0.040. While for the M9 model, the value of X^2^ (99) = 174.89, CFI = 0.983, TLI = 0.977 and RMSEA = 0.046, which shows good adjustment indices (See Table 3).

The metric invariance for the level of education and the method of administration of the questionnaire proved through the comparisons M6-M5 and M10-M9 respectively. The comparisons yielded ΔX^2^ = 18.05 (Δdf = 20; *p* = 0.584) and ΔCFI = 0.000 for the variance according to the level of education. While for the method of administration of the questionnaire, the comparisons generated ΔX^2^ = 2.38 (Δdf = 8; *p* = 0.967) and ΔCFI = 0.002 (See Table 3).

The scalar invariance for the level of education and the method of administration of the questionnaire proved through the comparisons M7-M6 and M11-M10 respectively. The comparisons yielded ΔX^2^ = 27.58 (Δdf = 20; *p* = 0.12) and ΔCFI = −0.002 for the scalar variance according to the level of education. While the comparison M11-M10 generated ΔX^2^ = 6.38 (Δdf = 12; *p* = 0.90) and ΔCFI = 0.001 (See Table 3).

Strict invariance across study level (M8-M7) and according to the administration of the questionnaire method (M12-M11) demonstrated a value of ΔX^2^ = 14.19 (Δdf = 24 at *p* = 0.942) and ΔCFI = −0.002 for the first invariance and ΔX^2^ = 14.31 (Δdf = 12 at *p* = 0.281) and ΔCFI = −0.001 for the second invariance (See Table 3).

As a conclusion, the factorial invariance of the measuring instrument was confirmed across the gender, the study level, and also the method of administration of the questionnaire.

Table 4 shows the results of correlations between the two dimensions of the PIPES scale with the measures of the IPAQ scale, the COVID-19 fear scale, and the two dimensions of the PSS10 scale.

**Table 4:** Pearson's correlation between the two dimensions of PIPES, the IPAQ, the CF-19 fear, and the PSS-10.

	**IPAQ**	**PIPES1**	**PIPES2**	**CF-19**	**Stress1**	**Stress2**
IPAQ	–					
PIPES1	0.328**	–				
PIPES2	−0.380^**^	−0.579^**^	–			
CF-19	−0.223^**^	−0.378^**^	0.331^**^	–		
Distress	−0.209^**^	−0.219^**^	0.226^**^	0.600^**^	—	
Coping	0.119^**^	0.008	−0.012	0.059	0.063	–

** *P < 0.01*.

A positive association between PIPES1 with IPAQ was demonstrated by a value of r = 0.328. While a negative correlation was found between the PIPES2 scale and the IPAQ scale. The IPAQ was able to explain 38% of the variance in the internal factor and 32.8% of the variance in the environmental factor of the PIPES.

Likewise, the results demonstrated a significant negative correlation between fear of COVID-19 and the PIPES1 scale (*r* = −0.378) and a moderate correlation with distress (*r* = −0.219). However, no link has been demonstrated between PIPES1 and the PSS-10 coping subscale.

For the link of PIPES2 with fear of COVID-19 and stress, the results showed a moderate positive correlation, on the one hand between PIPES2 and CF-19 (*r* = 0.331) and on the other hand between PIPES2 and general distress (*r* = 0.226).

## Discussion

The purpose of the present study was to develop and examine the psychometric properties of an instrument originally developed to measure perceived physical activity.

The reliability of the instrument examined in three ways showed that the two factors selected were consistent.

The results of the exploratory and confirmatory factor analysis and the factor invariance tests showed the robustness of the structure. The examination of configurational, metric, scalar and strict invariance confirmed the equivalence of the structure according to gender, level of education and mode of administration of the questionnaire.

Concurrent validity was tested by examining the association between the two factors of the instrument with the three scales: the IPAQ, COVID-19 fear, and perceived stress measured in two components.

The results showed that a negative perception of physical inactivity was positively associated with the IPAQ scale, and negatively associated with COVID-19 fear scores and perceived stress measured by Cohen's scale. Whereas positive perception of environment-related physical inactivity in COVID-19 was negatively associated with the IPAQ and positively associated with fear of COVID-19 and perceived stress. However, no association was found between coping strategies and the two components of the PIPES-10 scale.

To explain physical activity/physical inactivity, the two main models that have been put forward are the personality trait-based model and the ecological model.

The first model focuses on personality and will explain physical activity/inactivity by specific personality traits. For example, another study by Hoyt et al. (69) attempted to explain physical activity adherence through personality trait theory. They suggested that the traits of extraversion and activity awareness were associated with exercise behavior.

From the same perspective, Sutin et al. (70) studied the relationships between personality traits and physical inactivity in both sexes in several age groups. The results of their study concluded that lower neuroticism and elevated consciousness were linked to more physical activity and less physical inactivity. Furthermore, extraversion and openness were also associated with more physical activity and less inactivity.

Individuals who are rich in neuroticism (the tendency to feel negative emotions and stress) tend to avoid physical activity, while individuals who are rich in extroversion (the tendency to feel positive emotions and be outgoing) and conscience (the tendency to be organized and disciplined) tend to be more physically active (71). Openness to traits (the tendency to be open-minded and creative) has recently been associated with greater physical activity (72).

The second model addresses this issue in a system that integrates external factors to the individual, such as the environment, culture, politics, and society. Indeed, several studies have been able to establish the evidence of a great impact of the environment on personal choices in several contexts, such as participation in physical activity. Another parameter that favors the ecological approach is that it is possible to act on internal and external factors for the promotion of physical activity (73) while the personality traits are unchangeable in nature.

Several studies have supported the relationship between environmental characteristics and physical exercise. The results highlighted the relationship between physical practice such as infrastructure, adequate pedestrian walks, easy access to stores and services, access to recreational parks and public open spaces, and pedestrian accessible infrastructure, greenery and aesthetic landscapes, low crime rate, and sense of personal safety. Similarly, Liu et al. (74) linked access to physical activity infrastructure at work and home time spent on physical activity.

The ecological model attempts to explain participation in physical activity through the combination of internal individual factors such as beliefs, attitudes, and behavior (intra-individual) and individual factors such as environment, society, and culture (extra-individual) at the same time.

Moreover, on the one hand, there is a gap between perception and adherence to physical activity.

Much more, the perception of health itself can influence the perception of physical activity. As an example, in an exploratory work by Martinez-Harvell et al. (75) which aimed to identify predictors of adherence to physical activity in patients, the results showed that subjects with poor health, daily smoking, obesity, or kidney disease did not follow recommendations for physical activity.

On the other hand, in another study, Tuakli-WosorRowan and Gittelsohn (76) explored the links between perceptions of physical activity and physical activity behaviors with health factors among Ghanaian women using both qualitative and quantitative analysis. They concluded that physical activity barriers were associated with the time load that leaves no time for activity, family, and work obligations, as well as the absence of sports facilities. While the correct perception was related to weight loss, health issues and the top motivational factors for physical activity were “weight loss,” and “increased energy.”

However, specific interventions can affect the perception of physical activity. In this context, West et al. (77) explored the effects of a focus group session on behavior change in physical activity across subjects with a high risk for diabetes. They showed that the chat session helped improve the maintenance of physical activity.

During the COVID-19 pandemic, physically inactive people were considered by several authors to be at higher risk and the impact of the disease would be more severe.

Therefore, several global scientific recommendations have emphasized the major importance of maintaining optimal physical activity despite the security measures of quarantine and social distancing. In this regard, Hall et al. (38) classified physical inactivity and sedentary lifestyle as a persistent pandemic and aggravated by the containment measures taken during the COVID-19 pandemic period. Other researchers such as (78) even proposed physical activity as both a physical and mental therapeutic tool to withstand the negative consequences of quarantine during the pandemic.

Similarly, Jakobsson et al. (79) recommended that individuals maintain regular physical activity during self-isolation to prevent future chronic health problems due to sedentary behavior. They emphasized maintaining a minimum threshold of 150 min of moderate-intensity physical activity or 75 min of vigorous physical activity per week, as recommended by the World Health Organization as a health support solution (80).

This study makes some recommendations regarding physical activity practice.

### Conclusion and Recommendations

The present study developed an instrument to measure the perception of physical activity through two factors that have proven to be robust. The developed scale can be used as a tool for the perception of physical inactivity.

Examination of associations between PIPES scores with different background variables should be considered in future research. For example, the ease of access to physical activity and sports facilities, the safety of these structures in residential and professional areas can be linked to the perception of physical activity.

Also, future research must establish the links between daily time management and the time devoted to physical activity on the one hand, and the perception of PIPES physical activity and inactivity. Difficulty in time management, especially for people who have a job that requires a lot of time, can lead to a negative attitude toward physical activity.

Further person-centered studies could be conducted to categorize populations according to their perceptions of physical activity. this can lead to effective awareness campaigns that target vulnerable and at-risk people.

In future research, it is interesting to build measurement scales centered on both the perception of physical activity and the environment. Such an ecological approach can make it possible for us to measure the perception of physical activity that takes into account cultural and social specificities. This will facilitate the intervention for the promotion of physical activity.

### Limits of the Study

The first limitation concerns the study of the temporal stability of the two factors of the instrument, which could not be implemented in the present study.

Similarly, factorial invariance across different ages was not investigated, and it is very important to do so, especially for the elderly.

Although this study offers very interesting avenues for measuring perceived physical activity from an ecological perspective that takes into account the COVID-19 pandemic situation, it would be appropriate to expand the population and examine the psychometric properties of the instrument and its factorial invariance in other populations as well as to test for cultural differences.

It is important to note that examining the tool in specific populations such as those with chronic illnesses may contribute to the sensitivity of the instrument.

Finally, another limitation is the need to implement a review that addresses the relationship between perceived physical inactivity and environmental factors such as culture, policy and infrastructure specific to physical activity, and life safety.

## Data Availability Statement

The original contributions presented in the study are included in the article/supplementary material, further inquiries can be directed to the corresponding author/s.

## Ethics Statement

This work has received approval from the Ethics Committee of the Research Unit, Sportive Performance, and Physical Rehabilitation, High Institute of Sports and Physical Education, Kef, University of Jendouba, Jendouba, Tunisia and received ethical clearance from the UNESCO Chair Health Anthropology Biosphere and Healing Systems, University of Genoa, Genoa (Italy), the Higher Institute of Sport and Physical Education of Kef, Kef (Tunisia), and the Higher Institute of Sport and Physical Education of Sfax, Sfax (Tunisia). The proposal has been also approved by the Jendouba University Ethics Committee and was undertaken following the legal standards of the Helsinki declaration in 1964 and its corresponding amendments. Written informed consent to participate in this study was provided by the participants' legal guardian/next of kin.

## Author Contributions

NG and NB conceived the experiment. NG, NC, and NB collected and analyzed data. NG, NC, AT, LP, FA, and NB drafted and critically revised the paper. All authors contributed to the article and approved the submitted version.

## Conflict of Interest

The authors declare that the research was conducted in the absence of any commercial or financial relationships that could be construed as a potential conflict of interest.

## Publisher's Note

All claims expressed in this article are solely those of the authors and do not necessarily represent those of their affiliated organizations, or those of the publisher, the editors and the reviewers. Any product that may be evaluated in this article, or claim that may be made by its manufacturer, is not guaranteed or endorsed by the publisher.

## Abbreviations

CF-19: COVID 19 Fear
IPAQ: International Physical Activity Questionnaire
IPAQ-C, International Physical Activity Questionnaire (short: last 7 days)
PSS10: Perceived Stress Scale 10 items
MET: Metabolic Equivalent Tasks
PIPE S: Physical Inactivity Perceived Experience Scale
PIPES1: Physical Inactivity Perceived Experience Scale (Factor1)
PIPES2: Physical Inactivity Perceived Experience Scale (Factor1).
